# Impact of Obesity-Related Endoplasmic Reticulum Stress on Cancer and Associated Molecular Targets

**DOI:** 10.3390/biomedicines12040793

**Published:** 2024-04-03

**Authors:** Joud AlBashtawi, Hend Al-Jaber, Sara Ahmed, Layla Al-Mansoori

**Affiliations:** 1 College of Medicine, QU Health, Qatar University, Doha P.O. Box 2713, Qatar; ja2208629@student.qu.edu.qa; 2 Biomedical Research Center, Qatar University, Doha P.O. Box 2713, Qatar; haljaber@qu.edu.qa (H.A.-J.); s-ahmad-sh@hotmail.com (S.A.)

**Keywords:** endoplasmic reticulum, obesity, adipose tissue, cancer

## Abstract

Obesity, characterized by excessive body fat, is closely linked to endoplasmic reticulum (ER) stress, leading to insulin resistance and type 2 diabetes. Inflammatory pathways like c-Jun N-terminal kinase (JNK) worsen insulin resistance, impacting insulin signaling. Moreover, ER stress plays a substantial role in cancer, influencing tumor cell survival and growth by releasing factors like vascular endothelial growth factor (VEGF). The unfolded protein response (UPR) is pivotal in this process, offering both pro-survival and apoptotic pathways. This review offers an extensive exploration of the sophisticated connection between ER stress provoked by obesity and its role in both the onset and advancement of cancer. It delves into the intricate interplay between oncogenic signaling and the pathways associated with ER stress in individuals who are obese. Furthermore, this review sheds light on potential therapeutic strategies aimed at managing ER stress induced by obesity, with a focus on addressing cancer initiation and progression. The potential to alleviate ER stress through therapeutic interventions, which may encompass the use of small molecules, FDA-approved medications, and gene therapy, holds great promise. A more in-depth examination of pathways such as UPR, ER-associated protein degradation (ERAD), autophagy, and epigenetic regulation has the potential to uncover innovative therapeutic approaches and the identification of predictive biomarkers.

## 1. Introduction

Cancer has emerged as the second leading cause of death globally, stimulating extensive research in biomedical science to unravel its origins, tissue formation, and metastasis. It is responsible for approximately 14.1 million new cases and 8.2 million deaths annually [1]. Despite substantial investments and research efforts, cancer continues to pose challenges. Nevertheless, advancements at the cellular and molecular levels have enhanced our understanding of its etiology and spread, leading to continuous progress in cancer treatments [2,3]. While established risk factors for cancer include genetic predisposition, ionizing radiation, tobacco use, infections, unhealthy diet, alcohol consumption, sedentary lifestyle, and environmental exposures, obesity has emerged as a significant risk factor for various types of cancer. The prevalence of cancer is expected to rise due to the increasing occurrence of risk factors, particularly obesity and metabolic syndrome [3].

Adipose tissue normally plays a crucial role in regulating metabolic balance. However, metabolic disorders like obesity and type 2 diabetes often manifest with persistent inflammation and dysfunction in adipose tissue. Obesity, characterized by abnormal or excessive fat accumulation in adipose tissues, is considered a chronic inflammatory condition [4,5]. Previous studies have indicated that obesity-associated adipose tissue is exposed to various stressful conditions, including ER stress [6,7]. Interestingly, aberrant activation of ER stress sensors and their downstream signaling pathways have emerged as important regulators of tumor growth, metastasis, and response to cancer therapies such as chemotherapy, targeted therapies, and immunotherapy [8,9]. This review provides an in-depth examination of how ER stress triggered by obesity contributes to the initiation and advancement of cancer, along with the interplay between oncogenic signaling and various ER stress-related pathways in obese individuals. Furthermore, we explore potential therapeutic strategies aimed at addressing ER stress as a means of managing obesity. Additionally, we share our perspectives on approaches and future directions for modulating ER stress in the clinical treatment of obesity.

## 2. Endoplasmic Reticulum Structure and Functions

In 1902, Emilio Veratti made the initial discovery of the endoplasmic reticulum (ER) in muscle fibers, initially referred to as the sarcoplasmic reticulum, which was later recognized as comparable to the ER in other cells. Subsequently, Keith Porter visualized the ER using electron microscopy and named it the endoplasmic reticulum [10].

ER is a dynamic and expansive cellular structure with multiple functions, including calcium storage, protein synthesis, and lipid metabolism.

**Figure 1:**
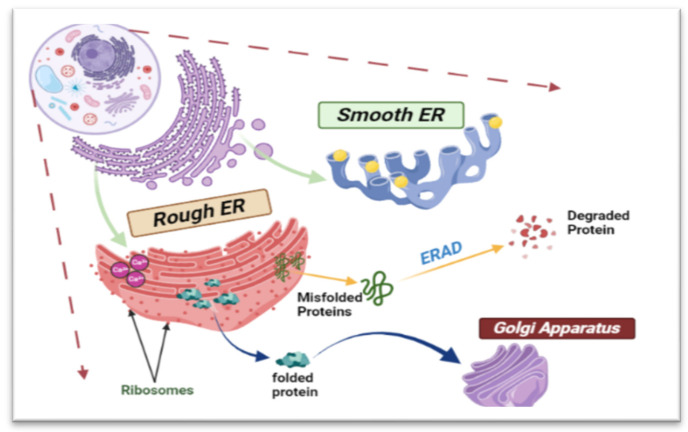
Schematic diagram showing the structure and functions of the ER. This complicated cellular organelle consists of interconnected tubules and sacs, with the rough endoplasmic reticulum (RER) housing ribosomes on its surface and the smooth endoplasmic reticulum (SER) lacking ribosomes. Misfolded proteins will be degraded through ER-associated degradation (ERAD).

The structure of ER is unique, comprising an outer nuclear envelope and a continuous membranous system known as the rough ER, along with a network of tubules and sheets known as the smooth ER (Figure 1). The dynamic morphology of the ER plays a significant role in protein biosynthesis. Additionally, the smooth ER is involved in lipid metabolism and enzyme detoxification [11].

### 2.1. Calcium Storage and Metabolism

The ER, a complex membranous network, crucially manages calcium storage and release, with calcium ions (Ca^2+^) as vital regulators of cellular processes [12]. The sarcoplasmic reticulum within the ER serves as the primary compartment for storing releasable calcium. The specialized region on the ER membrane, the sarcoplasmic/endoplasmic reticulum calcium ATPase pump (SERCA), plays a key role in maintaining cellular homeostasis by transporting Ca^2+^ from the cytosol to the sarcoplasmic reticulum [13,14]. Upon receiving a signal, functional membrane proteins, such as inositol trisphosphate receptors (IP3Rs) and ryanodine receptors (RyRs), are triggered to release Ca^2+^. The general design of these storage facilities appears to be planned to minimize any subsequent alterations, hence preserving the functional chaperones of luminal proteins [12].

**Figure 2:**
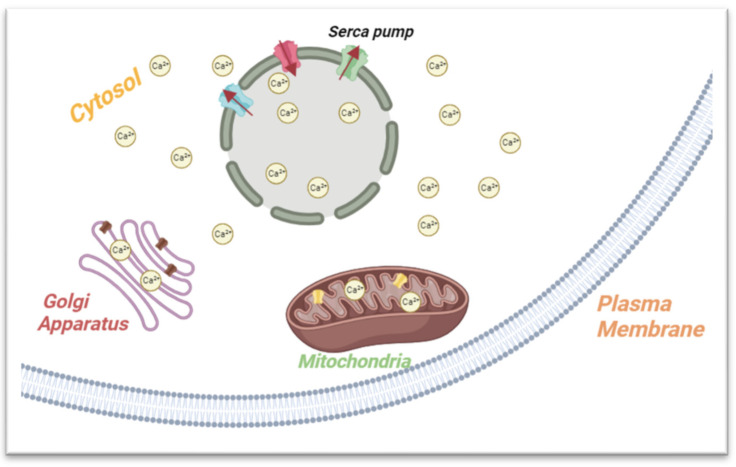
Summary of the ER’s role in calcium storage and metabolism. The ER membrane features SERCA, a crucial regulator maintaining cellular balance by moving calcium ions (Ca^2+^) from the cytosol to the ER, preserving low cytoplasmic calcium levels. After calcium release, restoration of resting levels occurs through SERCA, passive plasma membrane pumps, and organelle sequestration (e.g., mitochondria, Golgi).

Following the release of Ca^2+^, the ions should be removed to restore the resting calcium concentration to prepare for the next signal (Figure 2). This can be accomplished through active transport using the SERCA pump, passive transport via the plasma membrane calcium pumps, and sequestration into organelles like the mitochondria and the Golgi apparatus.

ER calcium storage significantly impacts cancer development, and understanding the complexities of Ca^2+^ transport and signaling in cancer cells holds promise for novel therapeutic strategies targeting calcium homeostasis [13]. Key points on ER calcium storage and cancer: (1) ER Ca^2+^ transporters: their aberrations in cancer contribute significantly to regulating ER Ca^2+^ stores; (2) Ca^2+^ signaling: this involves receptor isoform expression and downstream effector landscape3, often exploited in malignancies; (3) Ca^2+^-based therapeutic interventions: insights into ER Ca^2+^ transporters offer potential for therapeutic interventions; (4) mitochondria-associated membranes (MAMs): connectivity through MAMs is a primary mechanism for Ca^2+^ entry into non-excitable cells, modulated by intracellular Ca^2+^; (5) oncogenes and tumor suppressors: Ca^2+^ action centrality in carcinogenesis is linked to several oncogenes and tumor suppressors [13,14].

### 2.2. Protein Synthesis and Folding

**Figure 3:**
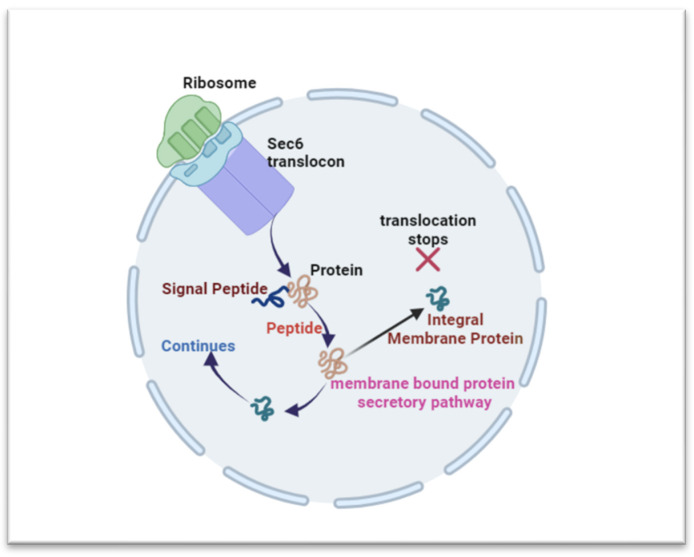
Overview of the ER’s involvement in protein synthesis and folding. The ER’s structural domains support protein production, with ribosomes on its surface. The canonical pathway anchors mRNA for translation initiation in the cytosol. Proteins are translated in the cytosol and continue in the ER via Sec6 translocon. A peptidase aids signal peptide cleavage, releasing the protein (cytosolic, integral membrane, and secreted proteins) into the ER lumen.

The ER’s membranous structures are involved in various cellular processes, with their primary functions being centered around the proper production, modification, and quality control of proteins. The ER’s several structural domains—each of which is linked to a particular function—enable it to produce cytosolic, integral membrane, and secreted proteins (Figure 3) [11].

The ER-driven protein synthesis involves ribosome arrangement, guided by the canonical pathway and co-transitional mRNA anchoring [11,15]. Translation initiates in the cytosol and shifts to the ER membrane through the Sec6 translocon, enabling the polypeptide’s ER entry [11,16]. Following translation, a peptidase cleaves the signal peptide, releasing the nascent protein into the ER lumen [11,17]. Proteins take distinct paths; integral membrane proteins halt translocation, while those for secretion enter the ER for modifications. Post-translation, proteins undergo activity, stability, and specificity-altering modifications [18,19]. Proteins for secretion undergo proper folding and modifications, guided by chaperones and enzymes, while those for ER function initiate folding. Misfolding triggers ER-associated degradation (ERAD) or retention in the ER [11,19].

ER protein synthesis plays a crucial role in cancer development, and its dysregulation can lead to the activation of the unfolded protein response UPR and the promotion of cancer cell apoptosis, as elaborated in the subsequent section. Understanding the mechanisms of ER protein synthesis and its connection with cancer development can help in the development novel therapeutic strategies [15,16].

### 2.3. Lipid Metabolism

ER lipid metabolism is a vital cellular process essential for maintaining cell structure and function [20]. It serves as the primary site for lipid synthesis, modification, and regulation, encompassing diverse molecules like fats, phospholipids, and sterols [20]. These lipids play key roles in cell membranes, energy storage, signaling, and cellular process regulation. The ER is centrally involved in lipid synthesis and distribution throughout the cell [21].

Dysregulated lipid metabolism is a key metabolic shift in cancer, marked by heightened lipid synthesis, storage, and uptake, contributing to cancer progression [17]. Tumor cells exhibit increased de novo lipogenesis, fatty acid uptake, and oxidation for energy and lipid accumulation. This dysregulation impacts membrane composition, gene expression, signaling pathways, and cellular functions, influencing cancer initiation and progression [18]. Lipid droplets (LDs) are crucial in cancer, serving as storage sites and regulating fatty acid flow and energy balance. Reprogramming lipid metabolism, including neo-lipogenesis and fatty acid oxidation changes, is critical in tumorigenesis [19] (C). Targeting the aberrant lipid metabolism presents a potential avenue for more effective cancer treatments.

### 2.4. Regulation of Lipid Metabolism

Lipid metabolism regulation encompasses pathways, such as the inositol-requiring enzyme 1 (IRE1), activating transcription factor 6 (ATF6), and pancreatic ER kinase-like ER kinase (PERK) pathways, which are pivotal elements of the UPR. These pathways play indispensable roles in maintaining cellular lipid equilibrium and shielding against biotoxicity [20]. IRE1, serving as a key UPR sensor, orchestrates cellular responses to the ER stress by controlling genes associated with lipid biosynthesis and metabolism [21]. PERK, another UPR sensor, influences lipid metabolism by modulating global protein synthesis and facilitating the activation of the transcription factor 4 (ATF4)-mediated transcription of genes linked to lipid regulation [22]. ATF6, the third UPR sensor, relocates to the nucleus during ER stress, activating genes responsible for lipid synthesis and adaptive responses [23]. Collectively, these pathways ensure the precise lipid balance within cells, guarding against cellular dysfunction stemming from lipid imbalances [20].

#### 2.4.1. IRE1

The IRE1α/X-box binding protein 1 (XBP1) pathway activates lipid-related genes like Lipin genes, *Osbp*, *Pecr*, *Lss*, and *Gpat4*, influencing lipid synthesis [24]. *XBP1* primarily impacts lipogenesis gene translation, crucial during liver transitions between fasting and refeeding [20]. Liver-specific *Xbp1* knockout disrupts fatty acid and sterol synthesis, lowering plasma triglyceride levels. *XBP1* contributes to ER expansion, influencing phosphatidylcholine and phosphatidylethanolamine synthesis. It aids in metabolic adaptation during fasting and ketogenic diets, with tissue-specific effects. Regulated IRE1α-dependent decay (RIDD) adds complexity to lipid metabolism, with outcomes shaped by the interplay between *XBP1* and RIDD activities, leading to context-dependent effects [25,26,27,28,29,30].

#### 2.4.2. PERK

*PERK* signaling, crucial for lipid balance, involves downstream components (eIF2α, ATF4, CHOP) and sensitive Insig-1, initiating lipogenesis. Absence of PERK in mammary cells reduces milk fat. Depleting Insig-1 in WT cells prompts SREBP processing via PERK and eIF2α, hindered in *PERK* KO cells. Comparable outcomes occur during hypotonic and ER stress [31,32]. In HCMV-infected cells, Insig-1 and SREBP1 activation by PERK operates independently of eIF2α phosphorylation. Overexpressing BiP in mice diminishes ER stress and SREBP-1c cleavage, reinforcing the UPR’s role [33,34,35]. Liver-specific eIF2α dephosphorylation impacts gluconeogenesis and lipid synthesis, affecting fasting-induced low blood sugar and high-fat-diet-induced liver fat. Transient activation of *PERK*-induced lipo- and glycogenic pathways is observed. Phosphorylated eIF2α boosts *ATF4* translation, influencing TG homeostasis. *ATF4* KO mice on a high-carbohydrate diet show reduced hepatic and serum TG levels, improved glucose and insulin tolerance, and reduced lipogenic enzymes. *ATF4* deficiency protects against diet-induced liver steatosis by reducing hepatic lipogenesis without impacting TG secretion and FA oxidation, shielding against fructose-induced hepatic hypertriglyceridemia [36,37].

#### 2.4.3. *ATF6*

*ATF6* regulates lipid metabolism independently of *XBP1*, enhancing choline kinase activity and phosphatidylcholine synthesis in NIH-3T3 cells. This effect, similar to *XBP1*, involves post-transcriptional or post-translational mechanisms [38]. ATF6 overexpression also increases *Acacb* and *Fasn* expression, enhancing fatty acid biosynthesis. In response to glucose deprivation, *ATF6* inhibits cholesterol synthesis by interacting with processed SREBP2, suppressing lipogenesis in HepG2 cells. Direct activation by sphingolipid intermediates triggers *ATF6’s* transcriptional program related to lipids. *ATF6’s* role in lipid metabolism is linked to PPARα, promoting fatty acid oxidation [39]. Moreover, *ATF6* deficiency or *dnATF6* overexpression in mice results in liver steatosis, while *ATF6* activation prevents triglyceride accumulation and enhances fatty acid oxidation. *ATF6* interacts with PPARα/RXRα heterodimers, crucial for their transcriptional activity in the liver, especially during fasting. Furthermore, *ATF6* plays a more critical role in promoting fatty acid oxidation than synthesis in the liver [40,41].

### 2.5. ER Homeostasis and ER Stress

ER is crucial for cellular functions, focusing on protein synthesis and folding. Disruptions, like unfolded proteins, trigger intricate responses: translation inhibition, selective degradation, and increased chaperones. Restoring ER function is vital for cell viability; any imbalance can lead to demise. ER stress, impacting functions like lipid and calcium metabolism, leads to abnormalities (Table 1). Persistent accumulation of misfolded or unfolded proteins overwhelms cellular quality control, resulting in ER stress. Exploring ER stress deepens understanding of strategies used by cells to preserve protein-folding balance, revealing their remarkable adaptability in challenging environments [42].

ER stress arises from unfolded or misfolded proteins in the ER lumen, triggered by infections, lipid accumulation, glucose deprivation, disturbed redox balance, or calcium regulation (Table 2) [5]. Persistent buildup of irregular proteins leads to cell apoptosis [6]. The body initiates the UPR to regulate new protein production and restore ER capacity, with ATF6, PERK, and IRE1, trans-membrane proteins in the lipid metabolism, influencing the UPR [6,45,46]. These proteins convey messages from the ER to the cytoplasm or nucleus, activating three pathways: (i) suppressing protein translation to prevent additional unfolded proteins; (ii) inducing ER molecular chaperone genes for protein folding; and (iii) activating ERAD to reduce unfolded protein accumulation. Unsuccessful attempts result in cellular imbalance and apoptosis [6,46,47,48].

**Figure 4:**
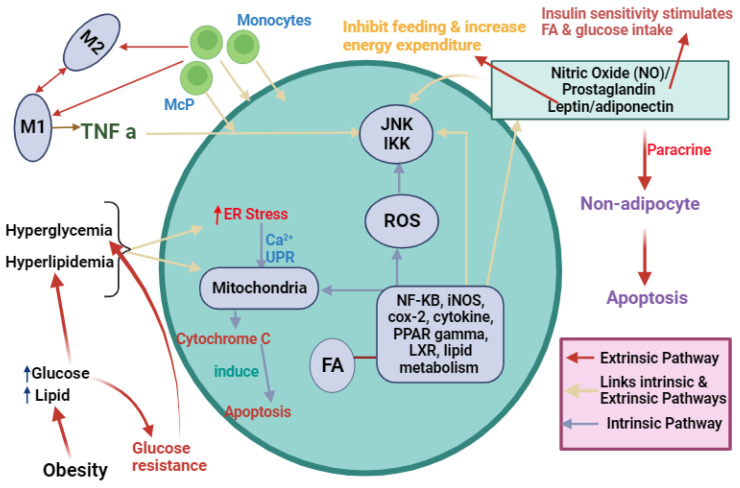
Outline of the key factors affecting adipocyte inflammation and metabolism leading to ER stress. Obesity induces hyperglycemia and hyperlipidemia, triggering ER stress through an intrinsic pathway leading to apoptosis. Another pathway (extrinsic) involving nitric oxide (NO), prostaglandin, and leptin/adiponectin activates JKK and IKK signaling, inhibiting feeding and increasing energy expenditure. Simultaneously, it triggers nuclear factor-κB (NF-KB), inducible nitric oxide synthase (iNOS), cyclooxygenase-2 (cox-2), cytokines, peroxisome proliferator-activated receptors (PPAR) gamma, liver X receptor (LXR), and lipid metabolism, culminating in paracrine signaling and adipocyte apoptosis. Monocyte differentiation into macrophages releases TNF-α, activating JNK and IKK pathways. This intricate network orchestrates adipocyte responses, linking extracellular cues to inflammation, metabolic changes, and apoptosis.

Obese adipose tissue exhibits enhanced adipocyte hypertrophy and hyperplasia, along with chronic inflammation involving inflammatory cell infiltration and cytokine network activation [6,49,50,51]. Recent studies link obesity to ER stress, which can trigger or result from obesity [52,53,54,55]. ER stress induced by obesity can lead to insulin resistance and type 2 diabetes, particularly in adipose tissues [56,57]. Elevated cytokines and free fatty acids in obesity signal c-JNK activation, implicated in inflammatory responses. In type 2 diabetes, JNK activation is associated with insulin resistance [58]. ER stress in obesity downregulates insulin receptor substrate-1 (IRS1) tyrosine phosphorylation (enhancing insulin signal inhibition) and upregulates serine IRS1 phosphorylation (inhibiting insulin signal) [57] (Figure 4). The IRE1 pathway, including XBP1, signals UPR-related gene transcription for protein folding and ER chaperones, limiting the ER stress response in cases of elevated XBP1 levels [57].

### 2.6. ER Stress and Cancer

Interestingly, emerging research has highlighted the refined connection between ER stress and cancer development. The complex interplay between ER stress and cancer involves both protective mechanisms aimed at restoring cellular homeostasis and pathways that can promote tumor growth and survival (Table 3). In tumor cells, ER stress may lead to the restoration of homeostasis and the creation of an environment that is favorable for the survival and growth of the tumor by releasing growth factors, cytokines, and angiogenic factors [59,60]. ER stress has the potential not only to impede cell apoptosis through its cytotoxic actions but also to promote tumor growth.

Unfolded protein response (UPR) induction is brought on by ER stress. Tumor cells exhibit both apoptotic and adaptive UPR. By increasing the secretion of vascular endothelial growth factor (VEGF), the adaptive action of UPR triggers anti-apoptotic NF-B, which blocks p53-dependent apoptotic signals and stimulates angiogenic activity. Moreover, IRE1, ATF6, and PERK, which are in the ER, are three ER-stress-signaling branches that are involved in carcinogenesis. The down-signaling XBP1 and IRE1 have a role in the development of cancer [76]. The progression of cancer is also aided by the PERK/eIF2/ATF4 branch of the ER stress pathway [77]. Separately, the ER-resident chaperone calreticulin, which is connected to both immunogenic cell death and the localization of calreticulin on the surfaces of tumor cells, has been localized to the cell surface in tumor cells. This connection might be linked to the production of ER stress in tumor cells.

Pancreatic cancer, a highly aggressive malignancy, has garnered significant attention due to its relationship with ER stress. The endoplasmic reticulum is a vital cellular organelle responsible for protein synthesis and folding, and disruptions in its normal functions can trigger ER stress. In pancreatic cancer, a combination of factors such as nutrient deprivation, oxidative stress, and an intense demand for protein synthesis can overwhelm the ER, leading to ER stress. This stress response has been implicated in various aspects of pancreatic cancer progression, including tumor initiation, growth, metastasis, and therapy resistance. The consequences of ER stress in pancreatic cancer involve complex cellular mechanisms, such as the UPR, which can either promote cancer cell survival or contribute to its demise. Pancreatic cancer proposes a very active UPR, which makes it resistant to therapy and promotes disease progression [50].

The three UPR receptors, IRE1, ATF6, and PERK, remain evident in pancreatic cancer. Glucose-regulated protein 78 (GRP78), also recognized as binding immunoglobulin protein (BiP), is an ER chaperone found in the ER’s lumen. It hinders the activation of *IRE1*, *PERK*, and *ATF6* by binding to their ER luminal segments. Accumulation of misfolded proteins within the ER, observed during instances of ER stress, leads to the separation of GRP78. This, in turn, triggers the activation of *IRE1*, *PERK*, and *ATF6,* along with their subsequent signaling pathways.

#### 2.6.1. IRE1

Inositol-requiring enzyme 1 (IRE1), a vital mediator of the UPR in the endoplasmic reticulum (ER), plays a crucial role in cancer development [51], regulating processes like phosphoinositide signaling lipids and macrophage growth. Key points on IRE1’s link to cancer include IRE1α and angiogenesis; IRE1α is a key regulator of angiogenesis, promoting blood vessel growth in tumors [52]. Moreover, IRE1 regulates phosphoinositide-signaling lipids crucial for cancer cell growth and survival [52].

#### 2.6.2. PERK

PERK, a pancreatic ER kinase, is vital in the unfolded protein response (UPR) within the endoplasmic reticulum (ER) and is linked to cancer development [53]. Despite this association, the direct link between PERK and cancer remains incompletely understood. Key points about PERK and its connection to cancer include PERK’s implication in regulating ER stress, which has links to cancer development [53]. ER stress can trigger the UPR, exhibiting both adaptive and cytotoxic effects on tumor cells1. The precise link between PERK and cancer is not fully elucidated; however, ER stress and the UPR can have both adaptive and cytotoxic effects on tumor cells. ER stress may either enhance tumor growth or induce apoptosis [54]. Moreover, obesity is associated with the development of ER stress, contributing to various diseases, including cancer [55]. However, the specific relationship between PERK and obesity remains unclear.

#### 2.6.3. ATF6

*ATF6* plays a crucial role in cancer development and has been found to regulate carcinoma progression through mTOR or PERK signaling pathways [47]. In the context of cancer and obesity, *ATF6* has been implicated in the development of obesity-related cancers. Some key points about *ATF6* and its association with cancer and obesity include the fact that *ATF6* has been found to promote cancer cell survival and proliferation by inducing endoplasmic reticulum stress, which, in turn, promotes autophagy and confers cancer cells’ resistance to chemotherapy treatment [47]. ER stress can induce the UPR, which can have both adaptive and cytotoxic effects on tumor cells [47]. Furthermore, obesity-induced ER stress causes chronic inflammation in adipose tissue, and the expression levels of ER stress markers were significantly up-regulated in obese mice. This suggests that *ATF6*, which plays a role in ER stress, may be involved in the development of obesity-related cancers. Additionally, *ATF6* has been found to play a significant role in the tumor microenvironment, promoting the growth of cancer cells and the development of metastases [56].

#### 2.6.4. GRP78

GRP78 is associated with obesity-related diseases and is also implicated in various aspects of cancer development, making it a potential target for therapeutic interventions in both obesity and cancer [57]. In the context of cancer, GRP78 is involved in several aspects of cancer development, including tumor survival, chemoresistance, angiogenesis, and metastasis. Many tumor cells overexpress GRP78 on the outer plasma membrane, and its expression has been correlated with tumor resistance, greater risk for cancer recurrence, and a decrease in patient survival [64]. Therefore, GRP78 at the cell surface has been proposed as a promising target for cancer therapeutics [64]. Additionally, covalent inhibition of GRP78 has been shown to disconnect the transduction of ER stress signals to inflammation and lipid accumulation in diet-induced obese mice, suggesting its potential role in anti-obesity effects [58].

## 3. Targeting Obesity Induced ER Stress Mediator for Cancer Therapy

Therapeutics targeting ER stress represent an emerging approach in the treatment of various diseases, particularly those associated with protein misfolding, inflammation, and cellular stress. Since ER is responsible for protein folding and lipid synthesis [78,79], when it becomes overwhelmed due to factors like an excessive buildup of unfolded or misfolded proteins, it triggers a stress response known as the UPR. Prolonged or severe ER stress can lead to cell dysfunction and cell death, contributing to the pathogenesis of several diseases.

Strategies directed at mitigating ER stress could offer a novel path for therapeutic interventions in addressing the disorder. For instance, there have been encouraging outcomes observed in treating the condition using methods like small molecule compounds, FDA-approved substances, and gene therapy, all with the goal of re-establishing ER balance. Furthermore, delving deeper into clinical trials and examining each distinct pathway involved, such as the UPR, ERAD, and autophagy, in the context of maintaining or restoring ER equilibrium, holds the potential to uncover innovative therapeutic approaches and possibly identify biomarkers that could predict clinical outcomes [79].

In the complex landscape of obesity-induced cancer, a multitude of promising therapeutic targets emerge, each holding the potential to alleviate the heightened cancer risk associated with obesity. Chief among these targets is IRE1α, a central component of the UPR pathway. Recent studies have yielded compelling evidence of IRE1α’s pivotal role in orchestrating the survival and proliferation of cancer cells within the obese microenvironment. Notably, a groundbreaking study conducted in this context concluded that targeting IRE1α not only disrupts the adaptive responses of cancer cells to ER stress but also enhances the efficacy of oncolytic therapies. This finding, marked by increased cancer cell vulnerability and improved treatment outcomes, underscores the critical importance of IRE1α as a therapeutic node [80].

Furthermore, another study was conducted that aims to decipher the role of UPR signaling in melanoma, a notoriously apoptosis-resistant skin cancer. It investigates whether heightened UPR pro-survival signaling results from increased ER stress due to genetic and environmental factors or whether it is actively induced by common melanoma-associated mutations. The study suggests utilizing ER stress to tilt UPR signaling toward apoptosis but acknowledges the need for precision to avoid undesirable outcomes. It advocates a multifaceted approach, combining agents inhibiting cytoprotective UPR signaling with those inducing ER stress, potentially adding metabolic interventions and immune activators to improve melanoma therapy. Ultimately, the study envisions harnessing ER stress for more effective melanoma treatment, with ongoing trials expected to further inform these strategies [81].

Expanding our scope beyond IRE1α, the roles of *PERK* and *ATF6*, also integral to the UPR, have garnered attention as promising targets in the battle against obesity-induced cancer. These pathways play pivotal roles in regulating the cellular stress response, and their precise modulation holds the potential to disrupt cancer cell resilience.

Moreover, molecular chaperones like GRP78/BiP, autophagy regulators, and ROS modulators are under scrutiny for their ability to hinder cancer cell adaptation and survival. Meanwhile, the intricate metabolic changes occurring during ER stress in the context of obesity are subjects of intense investigation, with researchers seeking ways to exploit these alterations for therapeutic benefit.

In parallel, the epigenetic landscape within obesity-related cancer initiation is being explored, opening new horizons for the modulation of epigenetic regulators as potential targets for intervention. The collective exploration of these diverse targets not only promises to revolutionize cancer treatment strategies but also offers hope to individuals navigating the complex interplay between obesity and cancer. As researchers continue to unravel the intricacies of these pathways and their potential for therapeutic manipulation, the future holds the promise of more effective and personalized approaches to countering obesity-induced cancer. 

## Figures and Tables

**Table 1:** ER Function in normal cells and the effects of ER Stress.

Normal Function of ER	Produced Molecule	ER Stress Impact on Normal Cellular Functions	Reference
***Lipid biosynthesis***	Phosphatidylcholine (PtdCho), phosphatidylethanolamine (PtdEtn), phosphatidylinositol (PtdIns), and basic sphingolipid structures	Overproduction of lipids will often lead to lipotoxicity and cause diseases such as insulin resistance and dyslipidemia	[24,25,43,44]
***Protein synthesis and folding***	Glycoproteins, soluble proteins, and membrane bound proteins	ER no longer synthesizes nor folds protein properly, leading to diseases such as ovarian cancer	[26,27,28,29,43]
***Calcium metabolism***	Calcium	Anti-apoptotic activity	[30,31,43]

**Table 2:** Common factors inducing endoplasmic reticulum (ER) stress.

ER Stress Inducers	ER Stress Stimulation Mechanism	Resulting Diseases	References
Accumulation of misfolded protein	Misfolded protein buildup and aggregation in the ER hinder normal cellular function and can be toxic, resulting in cell death.	Diabetes, Alzheimer’s disease, Parkinson’s disease, and Huntington’s disease	[32,33,34]
Changes in calcium homeostasis	Unfolded proteins build up due to changes in ER Ca^2+^ homeostasis, which, in turn, trigger ER stress and the unfolded protein response.	Diabetes mellitus, neurologic disorders, cancer, kidney disease, obesity	[30,34,35]
Nutrient deprivation	The development of respiratory super complexes (SCs) and mitochondrial bioenergetics are stimulated by ER stress and glucose restriction.	Protein energy malnutrition, scurvy, rickets, beriberi, hypocalcemia, osteomalacia, vitamin K deficiency, pellagra, xerophthalmia, and iron deficiency	[29,36]
Oxidative stress	Because the process of protein folding depends on redox equilibrium, oxidative stress can interfere with this process and increase the generation of misfolded proteins, which, in turn, increases ER stress.	Parkinson’s disease, asthma, rheumatoid arthritis, amyotrophic lateral sclerosis (ALS), multiple sclerosis, depression	[37,38,39]
Protein overexpression	Human cells trigger ER stress as a defense mechanism when the amount of unfolded protein exceeds the ER’s capacity to fold it (combine with protein accumulation).	Diabetes and neurodegenerative disorders	[40]
Viral infections	Viral infection triggers ER stress, which helps cells survive by preventing apoptosis. By encouraging the synthesis of C/EBP homologous protein (CHOP), some viral infections cause ER-stress-mediated apoptosis.	Encephalopathy characterized by spongiform changes, neurodegeneration, and gliosis, along with the pathogenic effects of viruses like hepatitis C virus, influenza virus, and herpes simplex virus, leading to associated diseases	[41]

**Table 3:** Cancer types and associated ER stress markers expression.

Cancer	ER Stress Markers Expressed	Oncogenic Process	Reference
Pancreatic	*PERK* encourages angiogenesis and boosts beta-cell insulinoma growth	Promote metastasis through the transcription factor CREB3L1	[61,62]
Ovarian	Overexpression of *Grp78*	*Grp78* induces tumor progression through the activation of JNKs and FAK1 pathways; it also regulates the tumor microenvironment, angiogenesis, and cell proliferation	[63,64]
Lymphoma	Under hypoxic conditions, *XBP1* splicing encourages tumor growth	XBP1 can induce epithelial–mesenchymal transition (EMT) and cell invasion and metastasis, activating the c-MYC signaling pathway	[42,45,65]
Colorectal	*ATF4* levels rise in extreme hypoxia	*ATF4* promotes tumor progression by activating the c-MYC signaling pathway, inducing epithelial–mesenchymal transition (EMT) and cell invasion	[46,47,48]
Breast	Elevated levels of protein and mRNA and Bip/Grp78 protein in severe hypoxia; *ATF4*-elevated levels of spliced mRNA XBP1, which favors the survival of the tumor	Grp78 initiates the activation of JNKs and FAK1 pathways; *ATF4* promotes EMT and angiogenesis	[48,64,66,67,68,69]
Prostate	Increased signaling of the *Grp78* to the cell surface	*Grp78* initiates activation of Akt and the PI3K/AKT oncogenic pathway	[70,71,72]
Liver	High levels of *Grp78*, which leads to the in vivo and ex vivo invasion of hepatocellular carcinoma	*Grp78* regulates PTEN-loss-mediated liver injury and cancer progression	[72,73,74]
Brain	Overexpression of *Grp78* decrease in XBP-1 will stimulate the viral oncolysis of the U373 glioblastoma cells	XBP1 is a transcription factor that promotes the expression of genes implicated in cell metabolism, nutrient uptake, and anti-oxidation	[49,74,75]
